# Tobacco smoke but not e-cigarette vapor induces epithelial barrier disruption, inflammation, and DNA damage in human Calu-3 cells

**DOI:** 10.1038/s41598-026-45438-9

**Published:** 2026-04-09

**Authors:** Bernd Mayer, Alexander Kollau, Wolfgang Kappaun, Katrin Rauchegger, Gerald Wölkart, Alexander Toedtling, Astrid Schrammel

**Affiliations:** Department of Pharmacology and Toxicology, Institute of Pharmaceutical Sciences, University of Graz, Humboldtstraße 46, Graz, A-8010 Austria

**Keywords:** e-cigarette vapor, Tobacco smoke, Claudin-1, DNA double strand breaks, Inflammation, Barrier integrity, Biochemistry, Cell biology, Diseases, Health care, Risk factors

## Abstract

Cigarette smoke is the leading cause of lung diseases including chronic bronchitis and chronic obstructive pulmonary disease (COPD). While harmful effects of cigarette smoke on lung epithelial function are well established, the consequences of inhalation of aerosols from e-cigarettes are less clear and disputed. In this study, human lung adenocarcinoma epithelial cells (Calu-3) were treated with extracts of cigarette smoke (CSE) or e-cigarette vapor (EVE) and comparatively analyzed for barrier function and integrity as well as for mRNA and protein expression of components forming the apical junctional complex (AJC). In addition, potential proinflammatory and genotoxic effects were investigated. Our results clearly demonstrate that CSE induced considerable disruption of the epithelial barrier leading to hyperpermeability and reduced protein expression of AJC proteins, especially of claudin-1. In addition, exposure of cells to CSE induced pronounced proinflammatory effects and increased DNA double strand breaks. By contrast, EVE, even if applied undiluted, neither affected functional nor structural parameters. In summary, on the basis of our results obtained with the Calu-3 in vitro model, we propose that e-cigarette vapor may exert less harmful effects on lung epithelial cells compared to cigarette smoke.

**Supplementary Information:**

The online version contains supplementary material available at 10.1038/s41598-026-45438-9.

## Introduction

The inhalation of tobacco smoke and other toxic gases disrupts the barrier function of the airway epithelium, facilitating the invasion of foreign substances and pathogens. The disruption of the lung epithelium barrier results in severe lung injury, in particular chronic obstructive pulmonary disease (COPD). Besides genetic susceptibility and diverse environmental factors, smoking is considered the main COPD risk factor. Oxidative stress caused by cigarette smoke is thought to disrupt the junctions between adjacent epithelial cells in the airways, leading to the recruitment of immune cells that trigger lung inflammation. The apical junctional complex (AJC) is a highly organized protein network that seals neighboring lateral membranes of polarized epithelial cells and controls paracellular permeability and epithelial homeostasis. It encompasses three significant proteins: claudin-1, occludin, and E-cadherin. Gene expression of these proteins is decreased by inhaled cigarette smoke through several mechanisms, including downregulation of protein kinase A, activation of epidermal growth factor receptor and downstream mitogen-activated kinase signaling, as well as activation of Rho kinase, resulting in impaired function of occludin and decreased E-cadherin expression.^1^.

While the harmful effects of cigarette smoke on epithelial barrier function are well established, the consequences of inhalation of aerosols from e-cigarettes are less clear. Clinical studies showed long-lasting significant improvement of COPD symptoms in smokers who abstained from smoking or had substantially reduced their cigarette consumption by switching to e-cigarettes^2^ or heated tobacco products (HTP)^3^. A prospective longitudinal study based on the data of Waves 1–5 of the U.S. *Population Assessment of Tobacco and Health* (PATH) study also indicated that the use of e-cigarettes did not significantly increase the risk of self-reported COPD incidents over five years if adjusted for smoking history of the respondents, while cigarette pack years remained associated with a net increase in COPD incidence risk^4^. However, the conclusions of this study were questioned in a Letter to the Editor^5^, and vaping is still considered a potential risk factor for COPD (see, for instance^6^.

In contrast to clinical observations, several animal and in vitro studies suggest that e-cigarette vapor may facilitate the development or progression of COPD. A recent study showed that mice exposed to e-cigarette vapor displayed enhanced emphysema and mucus accumulation, with increased immune cells in the bronchoalveolar lavage and increased levels of COPD-related cytokines in the lungs^7^. There is also evidence that the emissions of e-cigarettes may increase COPD risk through the promotion of oxidative stress in exposed tissues^8,9^. However, the levels of reactive species causing protein damage appear significantly lower than those observed in response to cigarette smoke^10^.

The available data on the effects of e-cigarette vapor on epithelial barrier function and on biochemical markers for inflammation are inconsistent. Several studies report on inflammatory and toxic effects on lung epithelial cells^11–13^ that may be mediated by nicotine^14^ or certain flavors^15,16^. Since these studies did not compare e-cigarette aerosols with tobacco smoke, the significance of the results for smokers switching to vaping products is unclear. Most studies reporting the effects of vapor and tobacco smoke found that barrier dysfunction was less pronounced upon exposure to e-cigarette aerosols^17–19^. Still, two studies found similar effects of smoke and e-cigarette vapor^20,21^.

In view of these conflicting results, we aimed to reinvestigate this issue *in vitr*o using human airway epithelial cells and directly compared cigarette smoke (CSE) and e-cigarette vapor (EVE) extracts for potential effects on epithelial barrier integrity, inflammatory status and DNA damage.

## Results

### Effects on epithelial barrier integrity

**Fig. 1:**
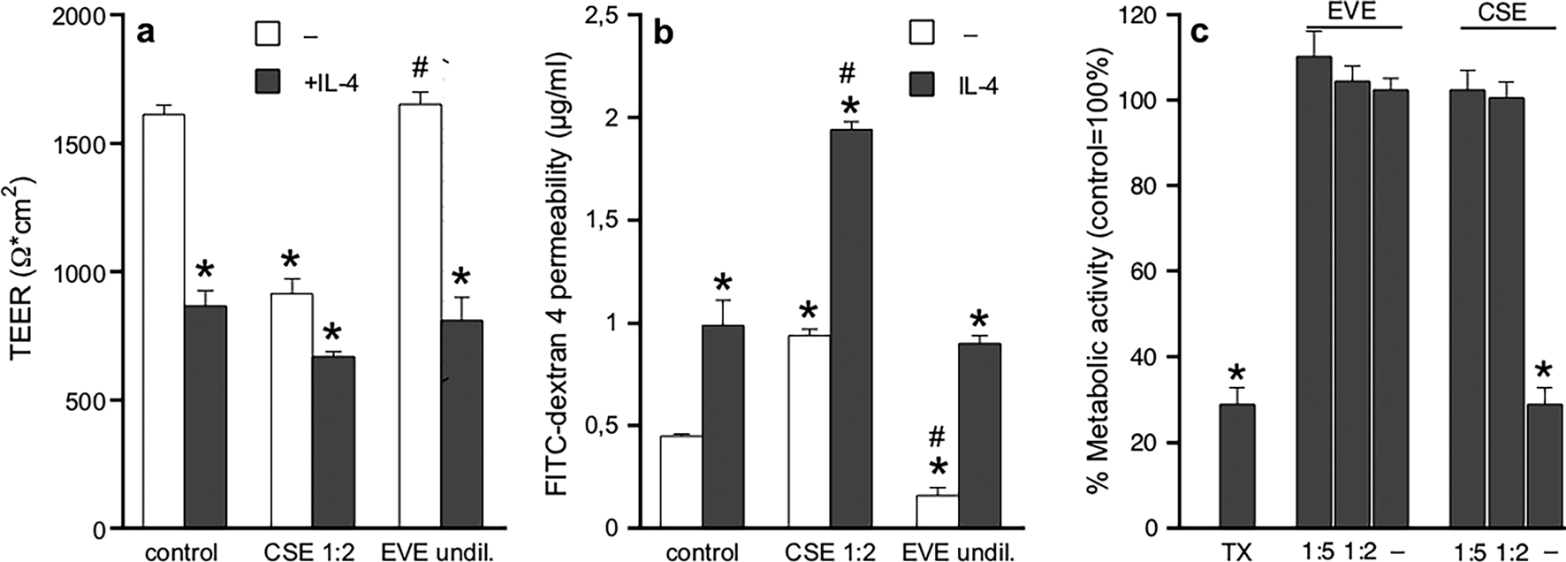
Effect of aerosol extracts on epithelial barrier function. (**a**) TEER was measured in monolayers of confluent Calu-3 cells under control conditions and in the presence of CSE (1:2) or EVE (undil.). Human IL-4 (5 ng/ml) served as positive control for induction of hyperpermeability. Data are expressed as mean values ± SEM of 6 individual experiments. *p < 0.05 vs. control, #*p* < 0.05 vs. CSE (ANOVA with Student-Newman-Keuls *post hoc* test). (**b**) Permeability of the epithelial barrier was measured using FITC-labeled dextran 4. Data are expressed as mean values ± SEM of 4 individual experiments. *p < 0.05 vs. control, #*p* < 0.05 vs. CSE (ANOVA with Student-Newman-Keuls *post hoc* test). (**c**) Cytotoxicity of extracts was measured in confluent Calu-3 cells by XTT test. Triton X-100 (1%; TX) served as positive control. Data are expressed as mean values ± SEM of 7 independent experiments obtained from 2 different cell batches. *p < 0.05 vs. control (Kruskal-Wallis test with Dunn´s *post hoc* test).

Potential effects of aerosol extracts on epithelial barrier function were studied in confluent monolayers of Calu-3 cells (TEER > 1500 Ω⋅cm^2^, Supplementary Fig. S1, S2), which represent an established in vitro model for studying airway epithelial biology. As shown in Fig. 1a, CSE (1:2; containing ∼80 µM nicotine) but not EVE (undiluted; containing ∼800 µM nicotine) significantly reduced TEER of the cell barrier compared to control cells. The effect of CSE was comparable to that of human IL-4 which served as positive control for induction of hyperpermeability^22^. In addition to TEER measurements, the integrity of the epithelial barrier was tested using fluorescein isothiocyanate (FITC)-labeled dextran 4 as molecular marker. As shown in Fig. 1b, an increase of the paracellular flux was observed in cell layers treated with CSE, corroborating the negative effect on barrier integrity by CSE. Interestingly, cell permeability was significantly reduced in the presence of EVE compared to control conditions suggesting even increased stability of the layer. Cell viability was measured in confluent Calu-3 cells by XTT test to ensure that the applied concentrations do not affect barrier function *via* cytotoxic reactions. While EVE showed no effect, undiluted CSE reduced metabolic activity by more than 70% (Fig. 1c). Therefore, CSE was diluted 1:2 in all experiments to avoid cytotoxic effects.

### Effects on apical junction complex assembly

**Fig. 2:**
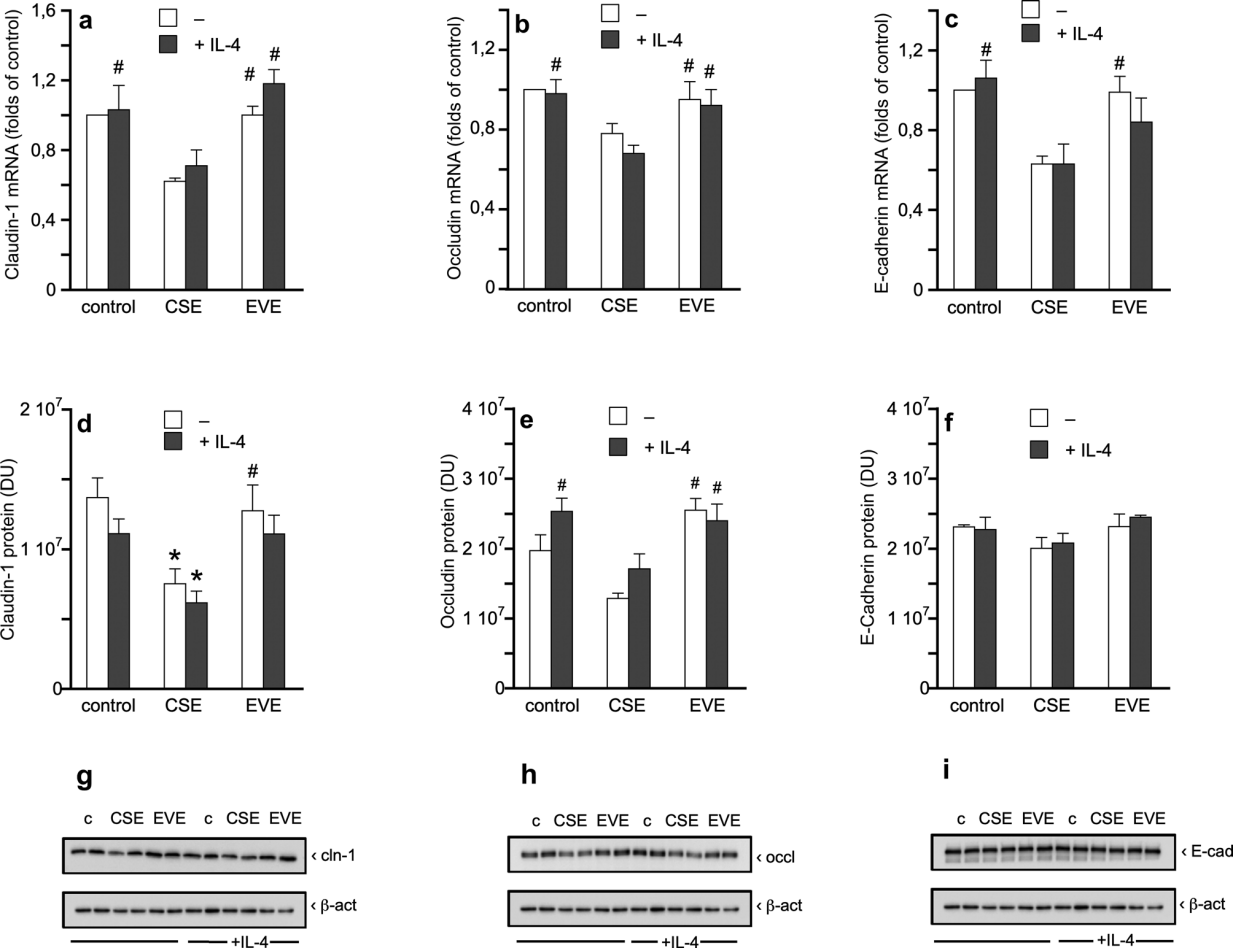
Effect of CSE (1:2) and EVE (undil.) on mRNA and protein expression of cell-cell interaction proteins. Experiments were performed with confluent monolayers of Calu-3 cells. Human IL-4 (5 ng/ml) served as positive control for induction of hyperpermeability. Results from qPCR experiments on expression of claudin-1 (**a**), occludin (**b**), and E-cadherin (**c**) represent mean values ± SEM of 3–6 experiments performed in duplicate and are expressed as folds of untreated controls (= 1). Protein expression of claudin-1 (**d**,** g**; cropped), occludin (**e**,** h**; cropped) and E-cadherin (**f**,** i**; cropped) was analyzed using β-actin (**g**,** h**,** i**; cropped) as endogenous control. Uncropped Western blots are shown in Supplemental figures S5–S8. Results from Western blot experiments represent mean values ± SEM of 4 single experiments. *p < 0.05 vs. control, #*p* < 0.05 vs. CSE. For **a**,** c**,** d**,** f**, ANOVA with Student-Newman-Keuls *post hoc* test, for **b**,** e**, the Kruskal-Wallis test with Dunn´s *post hoc* test was applied. cln-1, claudin-1; occl, occludin; E-cad, E-cadherin; β-act, β-actin.

To study the mechanism(s) underlying the effect of CSE on epithelial barrier function, mRNA and protein expression of TJ proteins claudin-1 and occludin as well as of the AJ protein E-cadherin were quantified under the experimental conditions applied for functional assays. At the mRNA level, a statistically significant reduction was observed for all targets in the presence of CSE when compared to EVE treatment (Fig. 2a-c). Likewise, protein expression of claudin-1 (Fig. 2d and g) and occludin (Fig. 2e and h) was significantly reduced after exposure to CSE compared to control conditions (-45% for claudin-1) and to EVE treatment (-41% for claudin-1; -50% for occludin). Interestingly, expression of E-cadherin was not affected at the protein level (Fig. 2f and i).

**Fig. 3:**
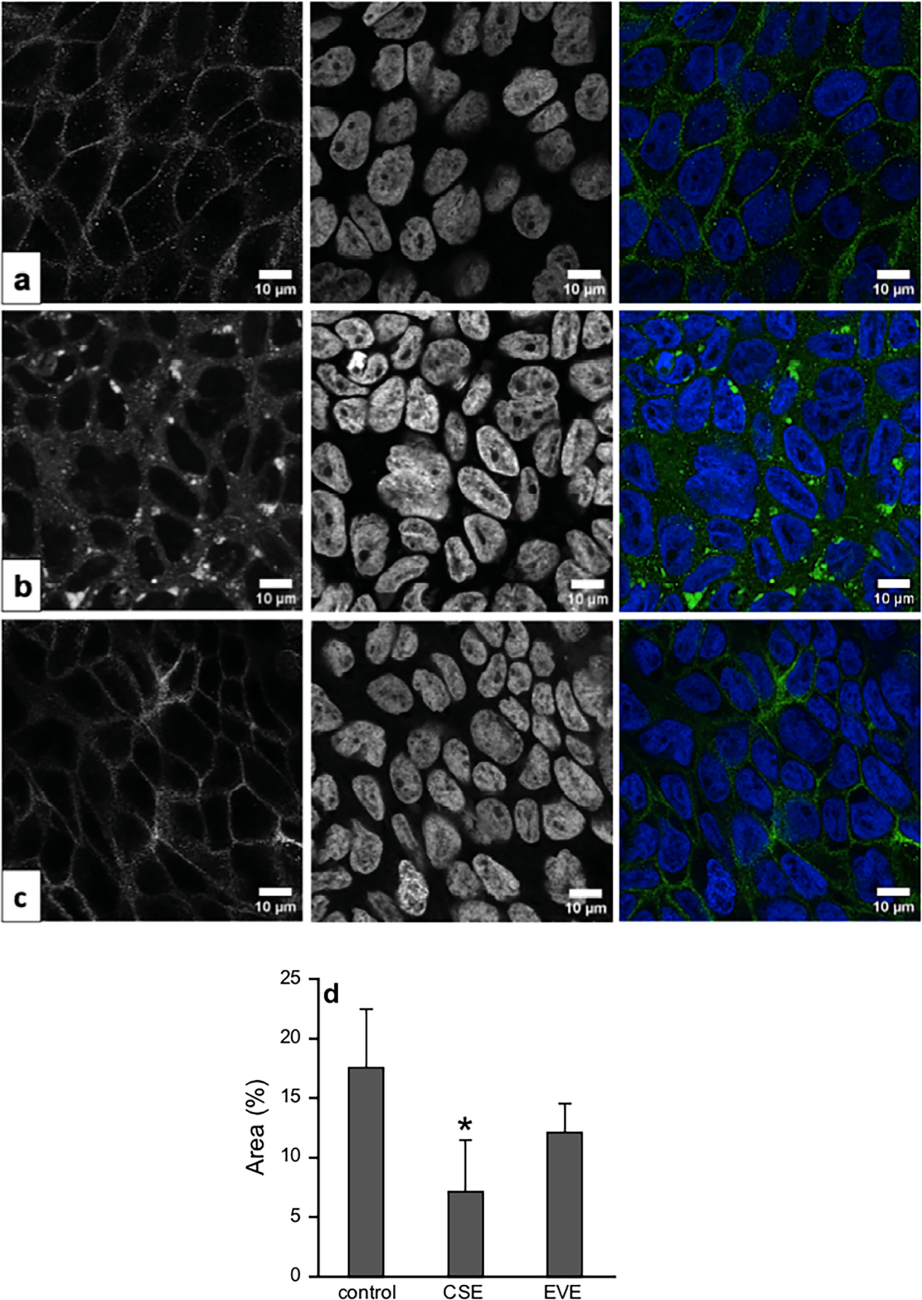
Effects of aerosol extracts on expression and localization of claudin-1. Expression of claudin-1 was visualized by confocal laser scanning microscopy (CLSM). Confluent Calu-3 cells were treated with vehicle (**a**), CSE (1:2) (**b**) or EVE (undiluted) (**c**) for 72 h, fixed with paraformaldehyde (PFA), permeabilized with Triton X-100, and counterstained with 4’,6-diamidino-2-phenylindole (DAPI) for *nuclei* (pseudocoloured blue). Claudin-1 was stained using an Alexa Fluor™ 488-conjugated secondary antibody (pseudocoloured green). From left to right: claudin-1 signal, DAPI signal for *nuclei*, merged, pseudocoloured image (**d**) Quantitative particle analysis of claudin-1 expression from Calu-3 cell micrographs was performed using Fiji image processing software. Data represent mean values ± SEM of 3 individual experiments. *p < 0.05 vs. control (ANOVA with Student-Newman-Keuls *post hoc* test).

Epithelial barrier function critically depends on the correct subcellular assembly of proteins forming the apical junctional complex (AJC), and disrupted barrier integrity leads to hyperpermeability which allows inhaled particles and pathogens to invade subepithelial layers. Considering the prominent effect of CSE on expression of claudin-1, we visualized its cellular localization by confocal laser scanning microscopy (CLSM). In Fig. 3, representative immunofluorescence stainings of claudin-1 are shown in cells treated with vehicle, CSE or EVE. Control cells (Fig. 3a) showed uniform and sharp signals in regions of plasma membranes that connect adjacent cells. By contrast, erratic and delocalized signals were detected in cells exposed to CSE (Fig. 3b). Moreover, clustering of signals was observed, indicating accumulation and/or aggregation of claudin-1. Cells treated with EVE (Fig. 3c) exhibited a staining pattern that mainly resembled that of control cells. Quantitative analysis of claudin-1 expression from micrographs using Fiji image processing software pointed to significantly decreased protein levels in cells treated with CSE compared to control cells (-51%), while EVE had no effect (Fig. 3d), strengthening the results obtained by Western blot analysis (Fig. 2d and g).

**Fig. 4:**
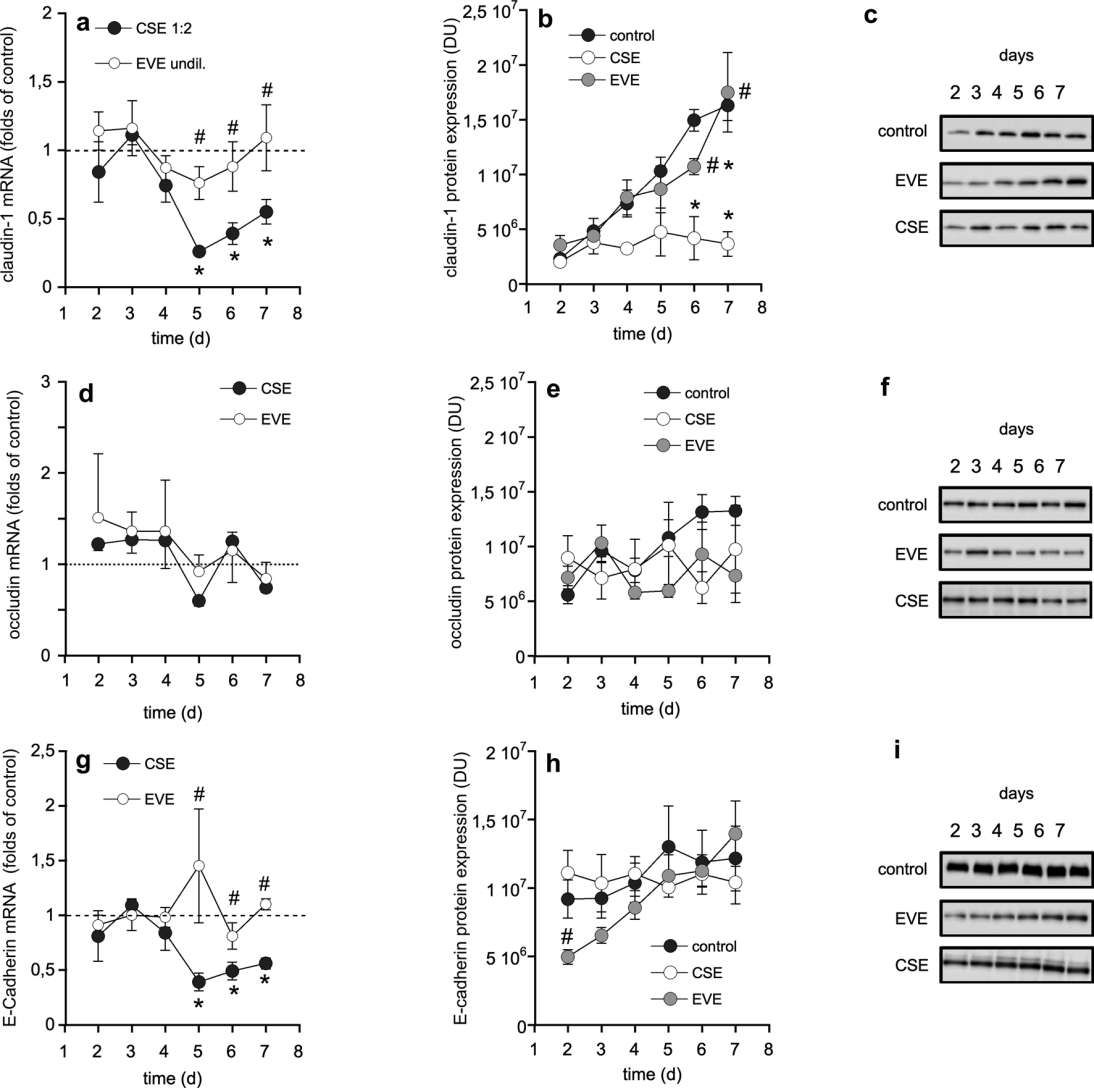
Effects of aerosol extracts on development of epithelial barrier. Calu-3 cells were treated with extracts (both diluted 1:2) in a non-confluent state (2.5 × 10^5^ cells per well) and mRNA and protein levels of claudin-1 (**a**-**c**), occludin (**d**-**f**), and E-cadherin (**g**-**i**) were daily analyzed over a period of 7 days by qPCR and Western blot analysis, respectively. Representative Western blots are cropped. Uncropped Western blots and Ponceau S stainings are shown in Supplemental figures S9–S11 (claudin-1), S12–S14 (occludin), and S15–S17 (E-cadherin). Data represent mean values ± SEM of 3–6 individual experiments (for Western blot) or duplicates (for qPCR). Results from qPCR experiments are expressed as folds of untreated controls (= 1). DU, densitometric units. *p < 0.05 vs. control, # *p* < 0.05 vs. CSE. For **a**,** b**,** d**,** e**,** g**, ANOVA with Student-Newman-Keuls *post hoc* test, for **h**, the Kruskal-Wallis test with Dunn´s *post hoc* test was applied.

To clarify the potential effects of CSE and EVE on the development of the epithelial barrier, Calu-3 cells were treated with extracts in a non-confluent state, and mRNA as well as protein levels were quantified daily (days 2–7). Expression of claudin-1 mRNA (Fig. 4a) and protein (Fig. 4b and c) was significantly reduced in the presence of CSE at days 5 (mRNA), 6, and 7 (mRNA and protein) compared to controls and EVE-treated cells. By contrast, occludin expression was not affected by CSE (Fig. 4d, e and f). mRNA expression of E-cadherin was significantly reduced on days 5, 6, and 7 in the presence of CSE compared to untreated and EVE-treated cells (Fig. 4g). However, this effect was not apparent at the protein level (Fig. 4h and i).

Effects of extracts on cellular protein levels and visualization of cell proliferation are shown in Supplementary Figs. S3 and S4, respectively.

### Effects on inflammatory status

**Fig. 5:**
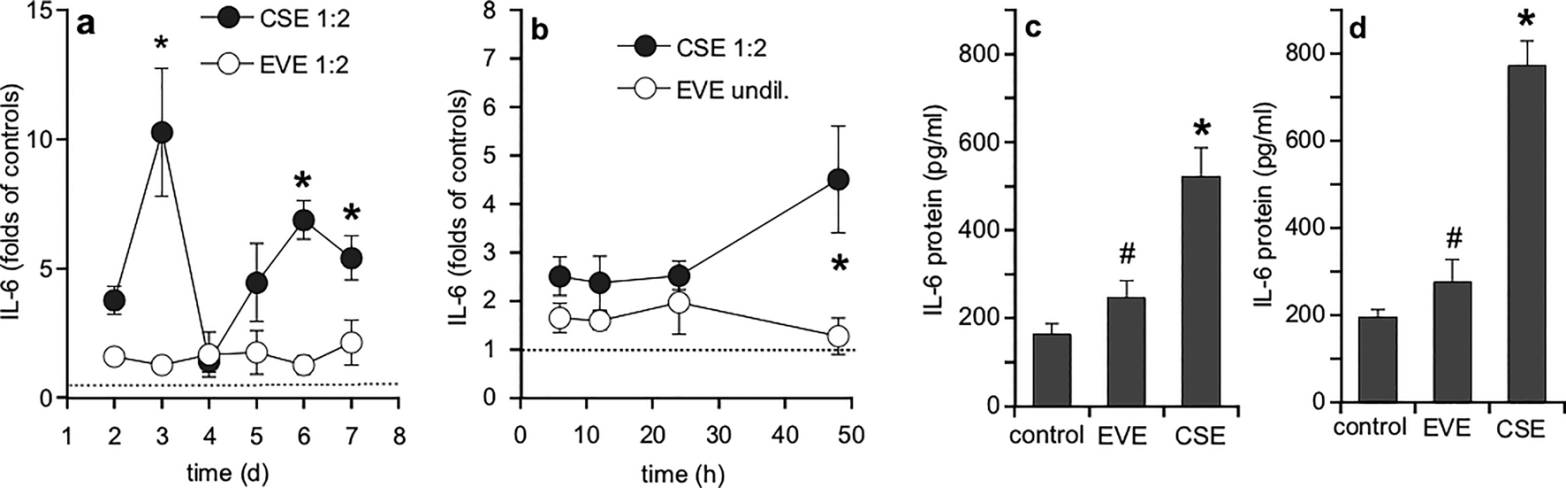
Effects of CSE (1:2) and EVE (undil.) on mRNA and protein expression of IL-6. (**a**) Calu-3 cells were treated with extracts in a non-confluent state and mRNA expression of IL-6 was daily analyzed over a period of 7 days. (**b**) Treatment of confluent cells with extracts was performed for up to 48 h. Results represent mean ± SEM of 5–6 experiments performed in duplicate and are expressed as folds of untreated controls (= 1). *p < 0.05 CSE vs. EVE (unpaired Student’s t-test). Secretion of IL-6 from confluent cells into the culture medium was measured by ELISA after 48 h (**c**) and 72 h **(d)**. Results represent mean ± SEM of 6 (48 h) and 3 (72 h) individual experiments, *p < 0.05 vs. control, #*p* < 0.05 vs. CSE (ANOVA with Student-Newman-Keuls *post hoc* test); *p* = 0,224 (control vs. EVE; 48 h); *p* = 0,449 (control vs. EVE; 72 h).

To test for proinflammatory effects of aerosol extracts, mRNA expression and protein secretion of IL-6 were measured after challenge of non-confluent and confluent cells with CSE and EVE. Under condition of a developing barrier, presence of CSE induced a peak of IL-6 mRNA concentration at day 3 (~ 10-fold compared to control cells), followed by a rapid decline at day 4 and a further significant increase at days 6 and 7 (Fig. 5a). Treatment of the mature, intact barrier with extracts for 48 h caused a ~ 4.5-fold increase of IL-6 mRNA in the presence of CSE (Fig. 5b). Presence of EVE did not affect IL-6 mRNA concentrations in both settings. As shown in Fig. 5c and d, IL-6 secretion into the medium was significantly increased ~ 3.5-fold and ~ 4-fold upon incubation of confluent cells with CSE for 48 h and 72 h, respectively, while EVE had no significant effect.

### DNA damage

**Fig. 6:**
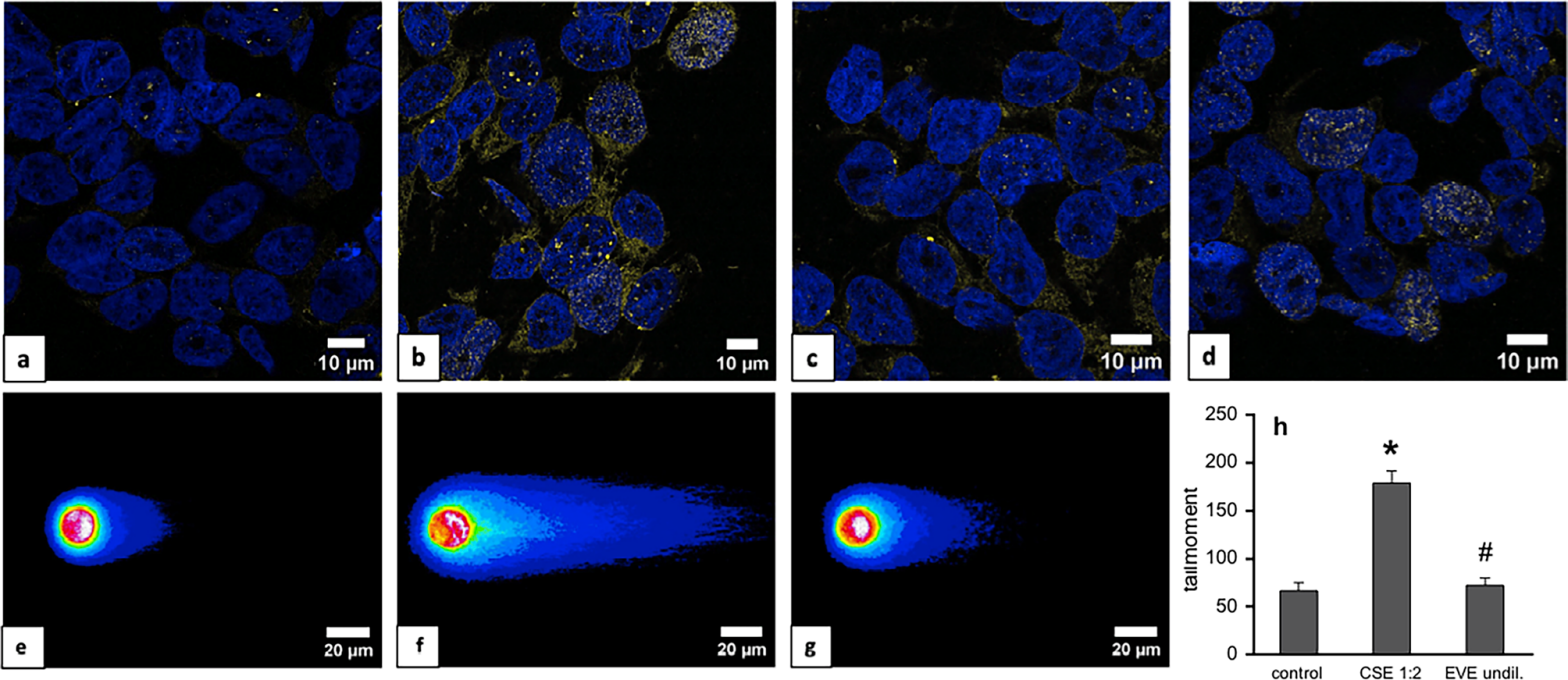
Visualization of DSB in the presence of aerosol extracts. Confluent Calu-3 cells were treated with extracts for 30 min, fixed with PFA and permeabilized with ice-cold methanol. Specimens were counterstained with DAPI for *nuclei* (pseudocoloured blue) and a phospho-H2AX antibody (pseudocoloured yellow) under control conditions (**a**) and after treatment with CSE (**b**), EVE (**c**), and etoposide (100 µM) (**d**). Quantification of DSB by neutral Comet assay. Calu-3 cells were treated with aerosol extracts for 30 min, suspended in low melting point agarose, sandwiched between microscope slides, lysed, and electrophoretically separated. Slides were then fixed with ethanol, stained with ethidium bromide, and visualized by fluorescence microscopy. Representative comets (pseudocoloured in 16 colours) are illustrated in **e** (control cells), **f** (CSE-treated cells), and **g** (EVE-treated cells). Scoring of the comet was performed with the OpenComet plugin and data are expressed as tail moment (6**h**). Data represent mean ± SEM of at least 30 individual cells for each condition obtained from 2 different cell batches. *p < 0.05 vs. control, #*p* < 0.05 vs. CSE (Kruskal-Wallis test with Dunn´s *post hoc* test).

To study potential DNA damage in cells by the extracts, phosphorylation of H2AX at Ser139 (γH2AX) was visualized by CLSM as a measure of nascent double strand breaks (DSB) in Calu-3 cells. As shown in Fig. 6, γH2AX *foci* (indicative for sites of nascent DSB) are represented as dots (pseudocoloured yellow) within the *nuclei* (pseudocoloured blue). Under control conditions (Fig. 6a), marginal staining was detectable. By contrast, cells exposed to CSE showed pronounced accumulation of γH2AX *foci* within *nuclei* (Fig. 6b). The intensity and the staining pattern were comparable to that of cells treated with the topoisomerase II inhibitor etoposide (100 µM), which is known to induce massive DNA damage and served as positive control (Fig. 6d). In the presence of EVE, less γH2AX *foci* were visualized within *nuclei*, indicating only moderate DNA damage (Fig. 6c). To quantify DSB, a neutral comet assay was performed, and DNA comets of control cells (6**e**), CSE-treated (6**f**), and EVE-treated cells (6**g**) were visualized by fluorescence microscopy and expressed as tail moment, a parameter that combines tail length and tail intensity in a single value. Analysis with the OpenComet plugin for Fiji software resulted in a ~ 2.7-fold increase in tail moment in the presence of CSE compared to control cells. On the contrary, there was no significant difference between control and EVE-treated cells (6**h**).

## Discussion

The aim of this study was to compare the effects of CSE with EVE on epithelial barrier function using a liquid/liquid interface model of human Calu-3 cells. Our results clearly demonstrate that CSE induced disruption of the barrier leading to hyperpermeability and altered protein expression of TJ proteins, especially of claudin-1. In addition, exposure of cells to CSE provoked pronounced proinflammatory and genotoxic reactions. By contrast, EVE did not significantly affect these parameters, even if applied undiluted. As the nicotine concentration was about 10-fold higher in EVE than in CSE, the observed effects of CSE were not caused by nicotine but other toxic compounds present in tobacco smoke.

In a previous study from Tatsuta et al., the effects of CSE exposure on barrier function and on expression of AJC proteins (claudins, occludin, *Zonula occludens* (ZO)-1, E-cadherin) were studied in an air-liquid interface setting using Calu-3 cells^23^. The authors proposed that CSE exposure leads to barrier dysfunction by downregulation of gene expression of multiple TJ and AJ proteins. The results of our study agree well with the findings of Tatsuta et al., both with respect to functional parameters (TEER and permeability) and with respect to gene expression.

Our experiments point to claudin-1 as the most vulnerable target of CSE. Among the 27 isoforms of the claudin family, claudin-1 is ubiquitously expressed and assumed to play a general role in epithelial barrier function^24^. From the mechanistic perspective, we propose a direct action on gene expression by components of CSE, but additional effects contributing to dissociation of claudin-1 from the AJC cannot be excluded. Thus, it is feasible that CSE also affects expression, structure, or function of cytosolic adaptor and scaffold proteins (e.g., cingulin and/or ZO-isoform), resulting in loosening of the AJC and eventually in the release of claudin-1 from the TJ assembly. Moreover, degradation of dysfunctional claudin-1 proteins or aggregates by the proteasome, endocytosis or lysosomes may contribute to attenuated claudin-1 protein levels in the presence of CSE. This hypothesis is supported by a recent study, showing that polyubiquitination of claudin-1 by an E3 ligase stimulates turnover of TJ in epithelial cells^25^.

The observation that CSE reduced mRNA and protein expression of claudin-1 during barrier development strengthens the notion of claudin-1 as most sensitive target. Formation of a mature and functional AJC is a complex, spatially and temporally confined process that starts with Rac-1-controlled *de novo* formation of AJ *via* dimerization of E-cadherin from apposing cells. Thereafter, Rho/ROCK activation triggers *de novo* formation of TJ with ZO-1 as central molecular driver^26^. In our experiments, negative effects on claudin-1 expression were observed 5–7 days after exposure to CSE, pointing to an interference of CSE compounds with molecular processes involved in later steps of AJC assembly. Ongoing work in our laboratory will clarify whether CSE exerts a direct effect on claudin-1 gene expression or indirectly interferes with the Rho signaling cascade that provides supply of polymerizable claudin for *de novo* TJ formation^27^.

The potent pro-inflammatory effects of CSE on the respiratory epithelium are well documented and ascribed to production of various cytokines and chemokines such as TNF-α, IL-1β, IL-6, and IL-8. This is consistent with our findings of significantly increased expression of IL-6 mRNA and protein levels. However, reports on the effect of EVE on the inflammatory state are inconsistent and comparison with CSE treatment is often lacking. According to a review from Masso-Silva et al.^28^, increased IL-6 levels were observed upon EVE exposure in epithelial cells, but a few studies showed decrease or downregulation of IL-6. In a recent study, effects of CSE and EVE on several parameters including IL-6 and IL-8 release were compared in a 3D human respiratory tissue model (EpiAirway™). While the authors described cytotoxic, proinflammatory and genotoxic effects for CSE, they found no discernible effects of EVE on any of the parameters assessed. Albeit we used a simple 2D model for our study, our results confirm these findings^17^.

Under conditions of barrier development, the highly reproducible, time-dependent effect of CSE on IL-6 mRNA expression was unexpected. The observation that IL-6 mRNA concentration peaked at day 3, rapidly declined to control values at day 4 and then increased again may be explained by effects on mRNA stability. IL-6 mRNA is highly unstable with a reported half-life of 30 minutes^29^. Certain RNA-binding proteins, including regnase-1^30^ and Arid5a^31^ were identified to promote or prevent decay of IL-6 mRNA by binding to destabilizing AU-rich elements within the 3′ untranslated region^29^. Thus, it is feasible that the observed effect of CSE is due to temporarily altered mRNA stability during the complex process of barrier maturation.

DNA DSB are regarded the most severe form of DNA damage due to the limited capability of DNA repair systems to accurately correct these lesions. Thus, analysis of DSB (e.g., by neutral comet assay and γH2AX immunostaining) is a reliable and widely accepted approach to probe DNA integrity in response to potentially genotoxic compounds. Negative effects of cigarette smoke constituents on DNA structure and function are undisputed, but the impact of e-cigarette vapor is controversially discussed. Thus, Yu et al. observed increased formation of DSB together with increased rates of apoptosis and necrosis in a keratinocyte cell line and head and neck squamous cell carcinoma cell lines exposed to e-cigarette vapor^32^. By contrast, in a study with a human lung epithelial cell line (BEAS-2B), no significant effect on formation of DSB was measured in the presence of e-cigarette vapor (γH2AX immunostaining), while CSE generated a dose-dependent increase in DSB^33^. Our results with Calu-3 cells agree with the latter study. Using a comet assay for quantification, we observed significantly increased formation of DSB in the presence of CSE, while EVE had no considerable effect. These results were further supported by γH2AX immunostainings.

Our results confirm the detrimental effects of tobacco smoke on the function of lung epithelial cells and provide strong evidence for markedly reduced damage of the epithelial barrier by exposure to nicotine containing e-cigarette aerosols. However, several limitations of our study need to be considered. First, in vitro experiments with cultured cells do not necessarily reflect the exposure of the lung epithelium in vivo. Even though Calu-3 cells are widely used for studying inhalative toxicity they do not recapitulate the complexity of a physiological bronchial epithelium. Since a central topic of this study was to assess epithelial integrity and its modulation by CSE and EVE, the Calu-3 cell model was chosen based on its ability to form tight monolayers with high and reproducible TEER. In future studies we aim to establish epithelial-endothelial cell co-cultures (in vitro air-liquid interface) to mimic the blood-gas barrier more accurately and to test if the observed effects of CSE and EVE are transduced to endothelial cells potentially affecting endothelial biology and function. Possibly, macrophages will be introduced into the cell model for generation of 3D triple cocultures allowing better simulations of inflammatory states.

Another limitation of our study the experimental setup for preparation of extracts is not validated for toxicological studies (since it lacks flow/pressure sensors to monitor the exact puffing profile). However, we are convinced that the device can accurately and reproducibly execute the reported puffing profile. In a previous paper, nicotine concentrations of CSE and EVE were quantified with very low variability between different experiments^34^. Values were similar to those reported by others^35^, reinforcing the relevance of our findings. Additionally, spectroscopical analysis of extracts (320 nm) did not show differences between the preparations.

Finally, the effects of liquid extracts used in our study may differ from the effects caused by direct exposure to aerosols. It should be emphasized that the extracts were prepared from nicotine containing liquid without flavors. Certain flavors, in particular vanillin and the chocolate flavoring 2,5-dimethylpyrazine^15^ as well as cinnamon and menthol^11,36^ were reported to impair the integrity of the alveolar blood barrier. Taken together, the effects of flavors on the epithelial barrier function are not fully understood and the in vivo relevance of the available in vitro data is unclear^15^.

Despite several limitations, our in vitro findings demonstrate clear differences between cigarette smoke extract and e-vapor extract in their effects on epithelial barrier integrity. These cellular results are consistent with, but cannot substitute for, observations made in human studies reporting reduced epithelial damage when smokers switch from combustible cigarettes to vaping^2^. However, given the constraints of the present in vitro model, our data should not be interpreted as direct evidence of improved health outcomes in vivo. Future investigations should therefore extend these findings by employing advanced organotypic models to determine the long-term consequences of vaping on lung health. In addition, systematic evaluation of specific e-liquid constituents - including potentially harmful flavoring agents such as cinnamon - will be important to delineate their individual toxicological profiles.

## Methods

### Epithelial cell culture

Human Calu-3 adenocarcinoma cells were a kind gift from Dr. Paul Vesely (Diagnostic and Research Institute of Pathology, Medical University of Graz). Cells were cultivated in Gibco™ Dulbecco’s Modified Eagle Medium/Nutrient Mixture (DMEM/F-12) (1:1) including 10% fetal calf serum (FCS), 1% penicillin/streptomycin and 1% non-essential amino acids at 37 °C in 5% CO_2_ atmosphere and 80% humidity in a Heracell™ VIOS 160i incubator (Thermo Fisher Scientific, Vienna, Austria). Cell numbers were counted using a DeNovix CellDrop™ counter (Biozym Biotech Trading GmbH, Vienna, Austria). For experiments, different approaches were designed (Supplementary Fig. S1). In protocol a, 2.5 × 10^5^ cells were seeded onto wells of 12-well plates (avantor™ delivered by vwr™, Vienna, Austria) and grown for 24 h. Then, CSE and EVE containing media were diluted 1:2 and applied on day 1 and cells were harvested daily over a period of 6 days either in RIPA buffer (Thermo Fisher Scientific) containing ethylenediaminetetraacetic acid (EDTA, 2 mM), COmplete™ Protease Inhibitor Cocktail (Roche), and phosSTOP™ (Roche) or lysis buffer (Isolate II RNA Mini Kit, Bioline, London, UK) for Western blot analysis and RNA extraction, respectively. In protocol b, 2,5 × 10^5^ cells were seeded onto wells of 12-well plates and grown to confluence for about 5 days. Thereafter, CSE and EVE containing media were applied on the intact epithelial barrier and cells were further incubated for up to 48 h. Cells were harvested after 6, 12, 24 and 48 h for biochemical analysis as described. For protocol c, 2.5 × 10^5^ cells were seeded onto Corning^®^ Costar^®^ Transwell^®^ plates (∅ 12 mm; containing 0.4 μm polyester membrane inserts) and grown to confluence (TEER > 1500 Ω⋅cm^2^). Thereafter, cells were incubated in the absence and presence of aerosol extracts. After 48 h, IL-4 (5 ng/ml) was added to distinct samples for induction of hyperpermeability and cells were further incubated for 24 h. Then, cells were analyzed functionally (TEER, FITC-dextran assay) or harvested for biochemical analysis.

### Preparation of aerosol extracts

Aqueous extracts were prepared using established protocols^34,35^ by passing cigarette smoke or e-cigarette vapor aerosol through 30 ml of Calu-3 cell culture medium using a glass impinger. An Ugo Basile rodent ventilator Modell 6025 (100 ml cylinder/piston assembly) connected to the glass impinger acted as a puffing machine to ensure a constant puffing regime (15 puffs à 55 ml taken over 3 s in 30 s intervals, square wave profile). For adequate comparison of the effects, stroke rates, volume, and length of tubing was kept consistent for all experimental protocols. Nicotine concentration of CSE was ∼160 µM^34^. To generate CSE, commercially available Marlboro red cigarettes (containing 0.8 mg nicotine, 10 mg tar, Philip Morris) were used. Aqueous EVE was prepared using an Eleaf iStick 50 W battery with Aspire Nautilus X 2 ml tanks and Nautilus X atomizer heads (1.8 Ω; InnoCigs GmbH & Co KG, Germany) operated at 15.0 W. The battery was fully charged before use, and the tank was filled with pure unflavored e-liquid (i-steam; ROPA, Vienna, Austria) containing nicotine (20 mg/ml) in 50/50 (vol/vol.) glycerol/propylene glycol. Nicotine concentration of the resulting EVE was ∼800 µM^34^.

For generation of EVE and CSE, the same method (ISO 20768:2018 recommended for generation of e-cigarette aerosols) was applied. Since there already exist robust data about negative effects of CSE on epithelial barrier function (some of which were substantiated by us), CSE served as reference in our study. There are various smoking regimes and vaping protocols with differences in puff volume, puff duration, puff intervals, puff profiles available which might produce (rather slightly) different results for toxicologically relevant parameters (e.g., particles or organic compounds). However, these differences seem rather negligible compared to the variability of measurements in biological matrices. For generation of CSE, commercially available cigarettes were used instead of standardized reference cigarettes. The brand we choose is still the most consumed one on the global market. In addition, to our knowledge, there are no standardized e-cigarettes available, thus far. Therefore, the comparison of commercially available products meant for consumption by humans seems reasonable.

### Quantification of protein concentrations

Protein concentrations were determined using the Pierce™ BCA Protein Assay Kit from ThermoFisher Scientific according to the manufacturer’s instructions as described^37^. Samples (25 µl) and standards of bovine serum albumin (BSA) were incubated with 200 µl reagent for 30 min at 37 °C and thereafter absorbance was measured at 562 nm using a SPECTROstar^®^ Nano microplate reader (BMG LABTECH GmbH, Ortenberg, Germany).

### Determination of cell viability

Cell viability was assessed using the XTT Cell Proliferation Assay Kit (#10010200) from Cayman Chemical, purchased through Sanova Pharma GesmbH (Vienna, Austria) as described^37^. Calu-3 cells were seeded on 96-well plates and incubated for 24 h with the extracts diluted in DMEM, as indicated in the text and figure. Colorimetric reactions were carried out according to the manufacturer’s instructions and absorbance was measured at 450 nm using a SPECTROstar^®^ Nano microplate reader (BMG LABTECH GmbH, Ortenberg, Germany).

### TEER measurements

For measurements of TEER, cells were seeded onto Corning^®^ Costar^®^ Transwell^®^ plates (∅ 12 mm; containing 0.4 μm polyester membrane inserts) in 0.5 ml of cell culture medium (apical chamber). Then, 1 ml medium was filled into the basolateral chamber of the Transwell^®^ plates. At the indicated time points, TEER was measured with an EVOM2 epithelial voltohmmeter (World Precision Instruments, Sarasota, FL., USA) equipped with STX2 chopstick electrodes (sterilized in 99.5% ethanol). Electrodes were cautiously placed in the medium of the apical and basolateral compartments. TEER values were corrected for resistance of cell-free inserts and multiplied by the surface area (r^2^ = 1.12 cm^2^)^38^.

### Measurement of paracellular flux

To measure cell permeability, 0.1 ml of FITC-labeled dextran, 4 kDa (1 mg/ml in cell culture medium) was added apically to cell layers and 0.5 ml of fresh cell culture medium was transferred into the basal compartment of the Transwell^®^ plates.

After incubation of cells for 2 h at 37 °C in 5% CO_2_ atmosphere and 80% humidity, FITC intensity of the basolateral medium was measured using a SpectraMAX^®^ Gemini™ EM microplate reader (Molecular Devices, LLC., Ismaning, Germany). Spectra were recorded with an excitation wavelength of 488 nm and an emission wavelength of 525 nm. FITC concentrations were calculated using a standard curve of FITC-dextran 4 (0.001 mg/ml, 0.003 mg/ml, 0.005 mg/ml, 0.01 mg/ml and 0.05 mg/ml). Measurements were performed in duplicate.

### Interleukin-6 ELISA

Calu-3 cells were seeded onto wells of 12-well plates and grown to confluence. Then, aerosol extracts (CSE 1:2; EVE undiluted) were applied, and cells were incubated for up to 72 h. At the indicated time points, IL-6 protein concentrations were quantified in the cell culture medium using the Human Il-6 Uncoated ELISA (#88-7066, Invitrogen™) according to the manufacturer´s instructions.

### Western blot

After harvest of cells in RIPA buffer, lysates were prepared by repeated sonication on ice. Aliquots were denatured by boiling with 5-fold Laemmli buffer for 10 min at 95 °C. Samples containing 15 µg of protein were separated by sodium dodecyl sulfate polyacrylamide gel electrophoresis (SDS-PAGE) on 6% or 12% gels for 45 min at 180 V. Thereafter, proteins were transferred onto nitrocellulose membranes (0.2 μm) for 80 min (240 mA). After blocking with Tris-buffered saline containing Tween^®^ 20 (0.1%; v/v) and non-fat dry milk (5%) for 1 h (ambient temperature) membranes were incubated overnight at 4 °C with primary antibodies (Supplementary Table 1). After incubation of membranes with respective horseradish peroxidase-conjugated anti-rabbit or anti-mouse IgGs (1:5,000) immunoreactive bands were visualized using Western Bright™ ECL or Western Bright™ Quantum (Biozym, Vienna, Austria) and chemiluminescence was quantified with the Fusion SL Imaging System (VWR International GmbH, Vienna, Austria).

### Real-time qPCR

Total RNA was isolated using the Isolate II RNA Mini Kit from Bioline. RNA concentration and quality were measured using a Nanodrop 2000 spectrophotometer (Thermo Fisher Scientific). Thereafter, mRNA was reversely transcribed into cDNA using Luna Script Reverse Transcriptase SuperMix (New England Biolabs GmbH, Frankfurt, Germany). Real-time qPCR analysis was performed with 10 ng of cDNA template, SsoAdvanced Universal SYBR Green Supermix (Bio*-*Rad Laboratories GesmbH, Vienna, Austria) and primers of distinct targets (300 nM) (Supplementary Table 2) on a StepOnePlus™ Real-Time PCR System (Thermo Fisher Scientific). Cycling conditions were as follows: 2 min at 50 °C, 10 min at 95 °C, 40 cycles of 15 s at 95 °C and for 1 min at 60 °C. Primers were tested for doubling efficiency before usage in final experiments. Data were analyzed according to the 2^−∆∆Ct^ method using cyclophilin D as reference gene. Lack of amplification was verified in no-template controls.

### Fluorescence imaging of claudin-1

Calu-3 cells were cultivated on coverslips (∅ 13 mm). After reaching confluence, cells were incubated in the absence and presence of aerosol extracts for 72 h. Thereafter, extracts were aspirated, cells were washed with phosphate-buffered saline (PBS) and then fixed with freshly prepared 4% paraformaldehyde (PFA) for 15 min at ambient temperature. After washing with PBS, cells were incubated with 0.1% Triton X-100 for 10 min, washed 3x with PBS and then incubated with 1% BSA for 1 h (blocking). After washing with PBS and 0,1% Tween^®^ 20 (PBST), cells were incubated overnight with a monoclonal claudin-1 antibody. Cells were washed 3x with PBST and then incubated under protection from light with an Alexa Fluor™ 488-conjugated secondary antibody (ambient temperature). After washing 3x with PBST, 4’,6-diamidino-2-phenylindole (DAPI, 1 µg/ml) was added for nuclear counterstaining. Cells were washed 3x with PBST and analyzed by CLSM (Leica Stellaris 5, Leica Microsystems GmbH, Wetzlar, Germany) equipped with a 63x objective using Leica Immersion Oil Type F. For visualization an adjustable white-light laser, fixed diodes (excitation: 405 nm, 488 nm and 638 nm) and Leica HyD hybrid detectors were used.

### Visualization of DNA damage

To assess DNA damage, DSB were visualized by detection of the phosphorylated histone variant H2AX at Ser139, which represents an early and specific cellular response to induction of DNA DSB^39^. For experiments, Calu-3 cells were seeded on coverslips (∅ 13 mm) in 24-well plates and grown to confluence for approximately 5 days. Then, cells were treated with culture medium or CSE- and EVE-enriched media for 30 min. After removal of media, cells were washed 3x with PBS, then fixed with 4% PFA for 10 min on a shaker. After removal of PFA, cells were washed with PBS, permeabilized with ice-cold methanol for 10 min at 4 °C, blocked with 1% BSA in PBS for 30 min and then washed again with PBS. Immunofluorescence staining was performed using the OxiSelect™ DNA DSB Staining Kit (Cell Biolabs, Inc.) according to the manufacturer´s instructions using a phospho-H2AX primary antibody and a FITC-conjugated secondary goat anti-mouse IgG. Phosphorylation of H2AX was visualized by CLSM (Leica Stellaris 5, Leica Microsystems GmbH, Wetzlar, Germany) equipped with a 63x objective (HC PL APO 63x/1,40 OIL) using FITC compatible filter settings. DAPI served as counterstaining.

### Neutral comet assay

The protocol of the comet assay was adapted from previously published articles^40–42^. Briefly, VWR^®^ Superfrost microscope slides (#631–1551) were pre-coated with a 0.8% agarose solution and stored at ambient temperature to allow drying overnight. At ∼60–70% of confluence, Calu-3 cells were carefully washed with PBS and detached with trypsin (0.05%) and EDTA (0,02%). Enzymatic reaction was stopped by addition of culture medium and the cell suspension was split into 3 equal volumes, which were diluted 1:2 with aerosol extracts or culture medium (control). Cells were incubated at 37 °C for 30 min under occasional gentle agitation. Thereafter, cell suspensions (250 µl) were mixed with 1 ml of low melting point agarose and volumes of ∼100 µl of each mixture (∼6.400 cells) were transferred onto pre-coated slides, immediately covered with coverslips (24 × 25 mm), and then put on an icepack for 10 min for faster solidification. Coverslips were carefully removed, and slides were placed in a lysis solution containing 2.5 M NaCl, 0.1 M EDTA, 10 mM Tris, 1% Triton X-100, and 10% DMSO (pH 10) for 1 h (4 °C), followed by three washing steps with Tris-acetate-EDTA (TAE) buffer (pH 8.3) for 5 min. Slides were transferred to an electrophoresis tank (filled with TAE buffer) and electrophoresis was then performed at 21 V (0.5 V/cm) for 30 min. Slides were washed 3x with PBS, fixed with absolute ethanol 2x for 10 min each, and then air-dried overnight at ambient temperature. After addition of 50 µl of ethidium bromide (2 µg/ml), slides were analyzed using an Epifluorescence Microscope (Zeiss Axiovert 200 M, Germany, 40x objective) and a CoolSNAP fx-HQ CCD-camera (Visitron Systems GmbH, Puchheim, Germany).

### Image processing, analysis and quantification

Image processing was performed with Fiji software (accessed in 2022, version v1.54i). Additionally, plugins were installed for image analysis and quantification. Otsu’s threshold algorithm was applied for image binarization. For quantitative analysis of the comet assay, OpenComet v1.3.1, an automated plugin tool for ImageJ, was used (default settings). At least 30 images per condition were captured with a 40x magnification objective. As recommended, the orientation of comets along the horizontal axis was changed before analysis, and tail moment values (an indicator of DNA damage represented by fragments migrating out of the *nucleus* and forming a tail) were analyzed by multiplying the length of the tail by the proportion of total DNA present in the tail. Yellow-marked comets and small unspecific dots, that were incorrectly recognized as cells, were removed as recommended.

### Statistical analysis

Results are presented as mean values ± SEM of n experiments. Data were tested for normality and homoscedasticity using the Shapiro-Wilk test https://www.statskingdom.com/shapiro-wilk-test-calculator.html and the Levene’s test https://statskingdom.com/230var_levenes.html from the Statistic Kingdom online platform, respectively. If data were normally distributed, unpaired Student’s t-test was performed to compare two experimental groups Analysis of variance (ANOVA) with Student-Newman-Keuls *post hoc* test was used to compare more than two groups using Kaleida Graph software V 5.0. For non-normally distributed data, the non-parametric Kruskal-Wallis test with Dunn´s *post hoc* test was performed https://statskingdom.com/kruskal-wallis-calculator.html. Significance was assumed at *p* < 0.05.

## Supplementary Information

Below is the link to the electronic supplementary material.

Supplementary Material 1

## Data Availability

The original data generated in this study will be made freely available in the Zenodo repository under 10.5281/zenodo.15006237.
