# Supplementary material for: Tobacco smoke but not e-cigarette vapor induces epithelial barrier disruption, inflammation, and DNA damage in human Calu-3 cells
Source: Sci Rep. 2026 Apr 9;16:16748. doi: 10.1038/s41598-026-45438-9 (PMC13223236; doi:10.1038/s41598-026-45438-9)
Supplement: Supplementary file 1 — Supplementary Material 1 [file 41598_2026_45438_MOESM1_ESM.docx]

**Supplementary Information**

**Tobacco smoke but not e-cigarette vapor induces epithelial barrier disruption, inflammation, and DNA damage in human Calu-3 cells**

Bernd Mayer^1^, Alexander Kollau^1^, Wolfgang Kappaun^1^, Katrin Rauchegger^1^, Gerald Wölkart^1^, Alexander Toedtling^1^, and Astrid Schrammel^1*^

^1^Department of Pharmacology and Toxicology, Institute of Pharmaceutical Sciences,

University of Graz, Humboldtstraße 46, A-8010 Graz, Austria

**Table of contents**

Supplementary materials and methods……………………………………….……………2

Supplementary figures ……………………………………………………...……………..4

**
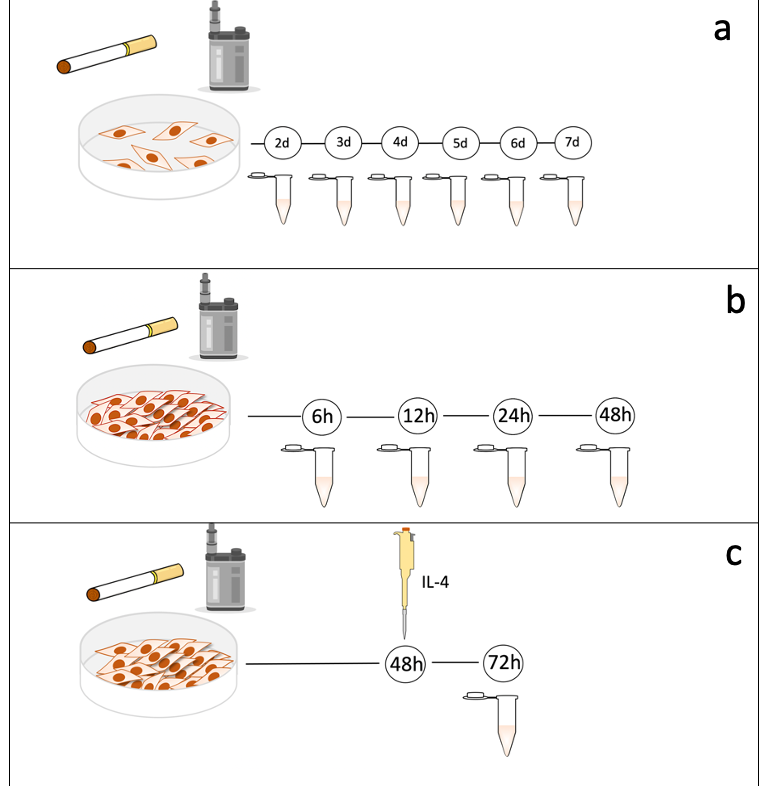
**

**Figure S1:** Experimental setup: (**a)** Cells were challenged with aerosol extracts in a non-confluent state (2,5 x10^5^ cells per well) and analyzed daily from day 2 to day 7. **(b)** Confluent Calu-3 cells (TEER >1500 Ω*cm^2^) were incubated in the absence and presence of aerosol extracts for up to 48 h. Samples were analyzed at indicated timepoints (6, 12, 24, and 48 h). (**c***)* Confluent Calu-3 cells were incubated in the absence and presence of aerosol extracts. After 48 h, IL-4 was added to distinct samples (for induction of hyperpermeability of the barrier) and cells were further incubated for 24 h. Then, cells were analyzed functionally (TEER, FITC-dextran assay) or harvested for biochemical analysis.

| **PRIMARY ANTIBODIES** | | | |
| --- | --- | --- | --- |
| **Antibody** | **Dilution** | **Company** | **Catalogue #** |
| Claudin-1 | 1:1000 (WB)  1:200 (IHC) | Cell Signaling | 13255 |
| Occludin | 1:1000 | Santa Cruz | 133256 |
| E-Cadherin | 1:1000 | Cell Signaling | 3195 |
| ß-actin | 1:200000 | Sigma | P1978 |
| **SECONDARY ANTIBODIES** | | | |
| **Antibody** | **Dilution** | **Company** | **Catalogue #** |
| Anti-mouse | 1:5000 | Cell Signaling | 7076 |
| Anti-rabbit | 1:5000 | Cell Signaling | 7074 |
| Anti-rabbit | 1:200 | Invitrogen | A11008 |

**Table 1**: List of antibodies (targets in alphabetical order)

| Claudin-1 | fw | 5’ CCGGCGACAACATCGTGAC 3’ |
| --- | --- | --- |
|  | rev | 5’ CGGGTTGCTTGCAATGTGC 3’ |
| Occludin | fw | 5’ TGCATGTTCGACCAATGC 3’ |
|  | rev | 5’ AAGCCACTTCCTCCATAAGG 3’ |
| E-cadherin | fw | 5’ GAAGGTGACAGAGCCTCTGGAT 3’ |
|  | rev | 5’ GATCGGTTACCGTGATCAAAATC 3’ |
| IL-6 | fw | 5’ ACTCACCTCTTCAGAACGAATTG 3’ |
|  | rev | 5’ CCATCTTTGGAAGGTTCAGGTTG 3’ |
| Cyclophilin D | fw | 5’ CAAGCATGATCGGGAGGGTT 3‘ |
|  | rev | 5’ TGTCGCCAGAGCCATCTTTT 3 |

**Table 2:** List of primers (targets in alphabetical order)

**Figure S2:** Time-dependent formation of the epithelial barrier monitored as increase of TEER under control conditions.

**Figure S3**: Effects of aerosol extracts on total cellular protein (protocol a).


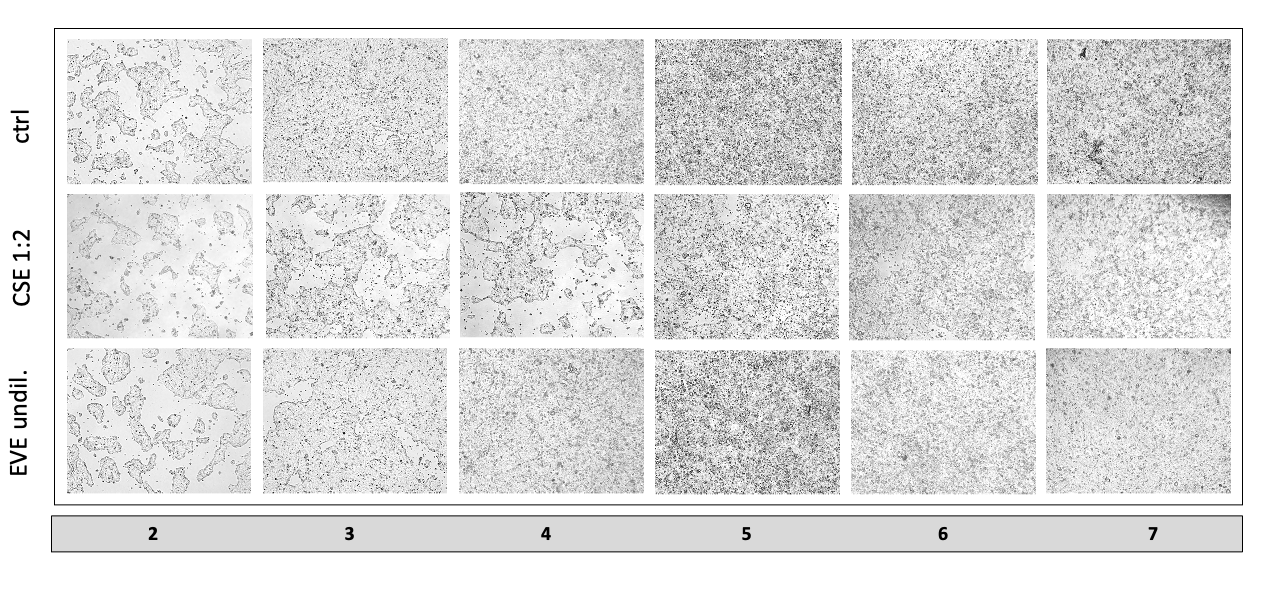


**Figure S4:** Effects of aerosol extracts on proliferation of Calu-3 cells (protocol a).

**Uncropped representative Westernblots**

—20 kDa

**Figure S5:** Uncropped Western blot of claudin-1 expression.

Area that is outlined in blue is shown in the upper panel of Fig. 2**g**.

—65 kDa

**Figure S6:** Uncropped Western blot of occludin expression.

Area that is outlined in blue is shown in the upper panel of Fig. 2**h**.

—135 kDa

**Figure S7:** Uncropped Western blot of E-cadherin expression.

Area that is outlined in blue is shown in the upper panel of Fig.2**i**.

—45 kDa

**Figure S8:** Uncropped Western blot of β-actin expression. Area that is outlined

in blue is shown in the lower panel of Fig. 2**g**, 2**h**, and 2**i**. Note, that all targets were

detected on the same representative blot; thus, the loading control (β-actin)

is identical in Fig. 2**g**, 2**h**, and 2**i**.

—20 kDa

**Figure S9a:** Uncropped Western blot of claudin-1 expression.

Area that is outlined in blue is shown in the upper panel of Fig. 4**c**.


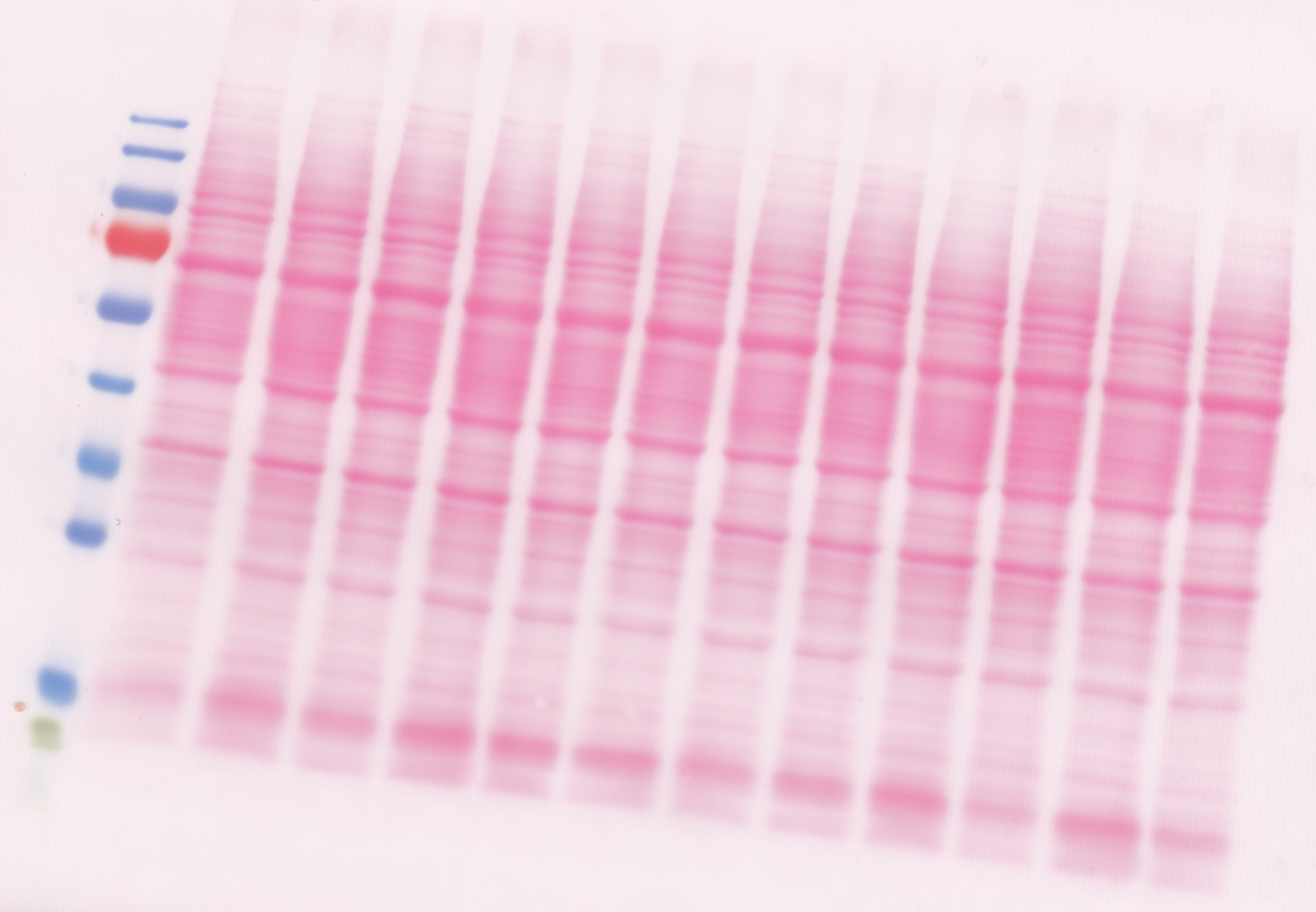


**Figure S9b:** Uncropped Ponceau S-stained membrane used for

detection of claudin-1 expression. (**Figure S9a)**.

—20 kDa

**Figure S10a:** Uncropped Western blot of claudin-1 expression.

Area that is outlined in blue is shown in the middle panel of Fig. 4**c**.

**
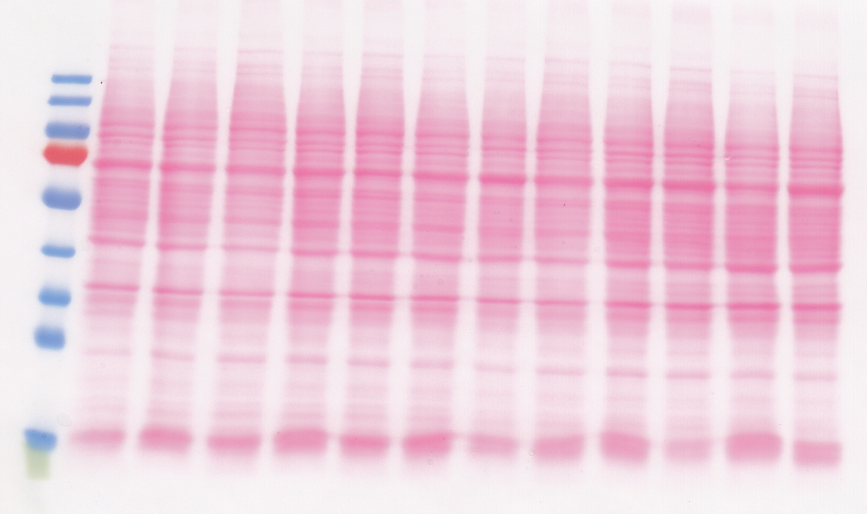
**

**Figure S10b:** Uncropped Ponceau S-stained membrane used for

detection of claudin-1 expression. (**Figure S10a)** Of note, the same

membrane was used for blots shown in **Figures S12a** and **S13a**.

—20 kDa

**Figure S11a:** Uncropped Western blot of claudin-1 expression.

Area that is outlined in blue is shown in the lower panel of Fig. 4**c**.


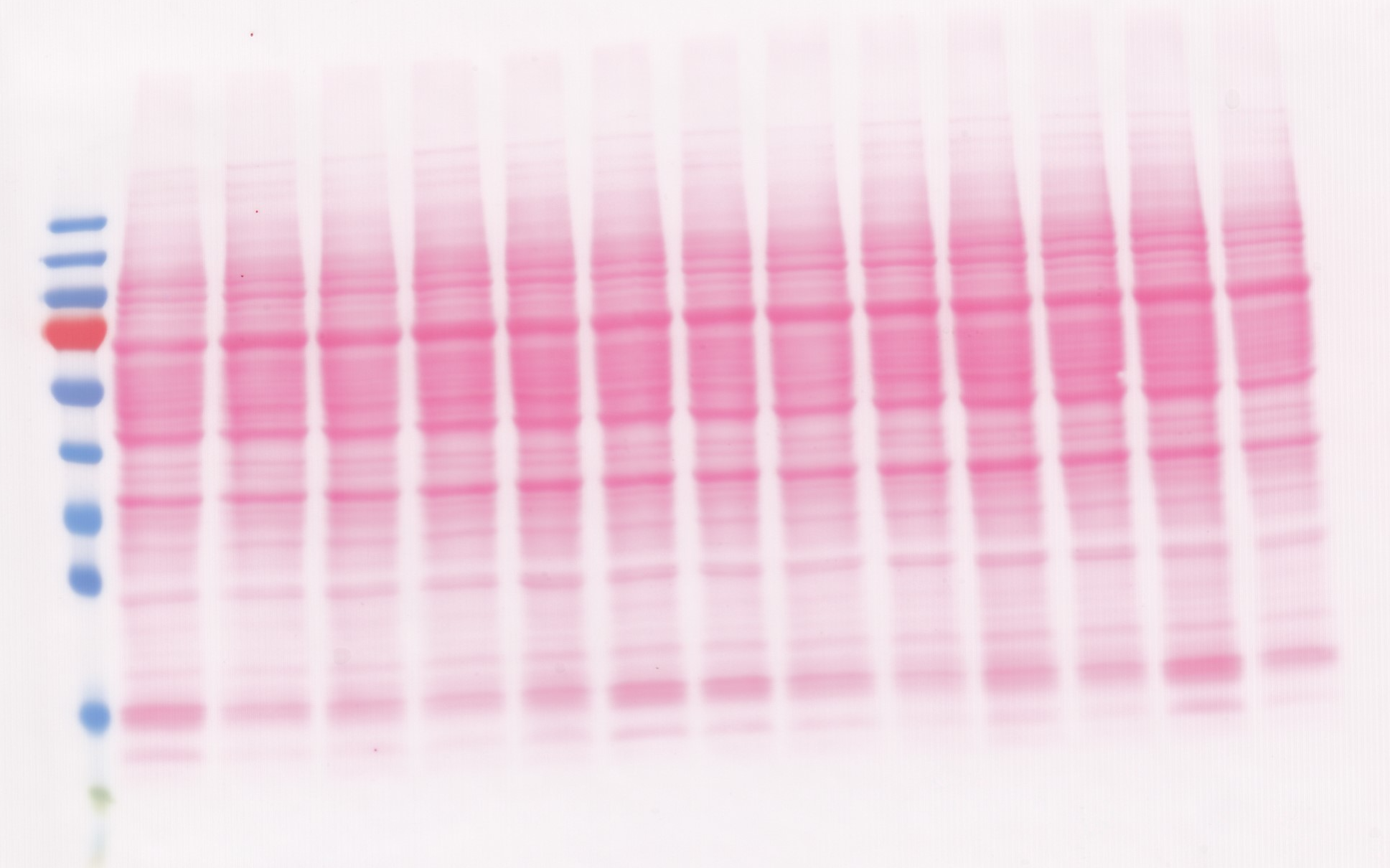


**Figure S11b:** Uncropped Ponceau S-stained membrane used for

detection of claudin-1 expression. (**Figure S11a)**.

—65 kDa

**Figure S12a:** Uncropped Western blot of occludin expression.

Area that is outlined in blue is shown in the upper panel of Fig. 4**f**.

**
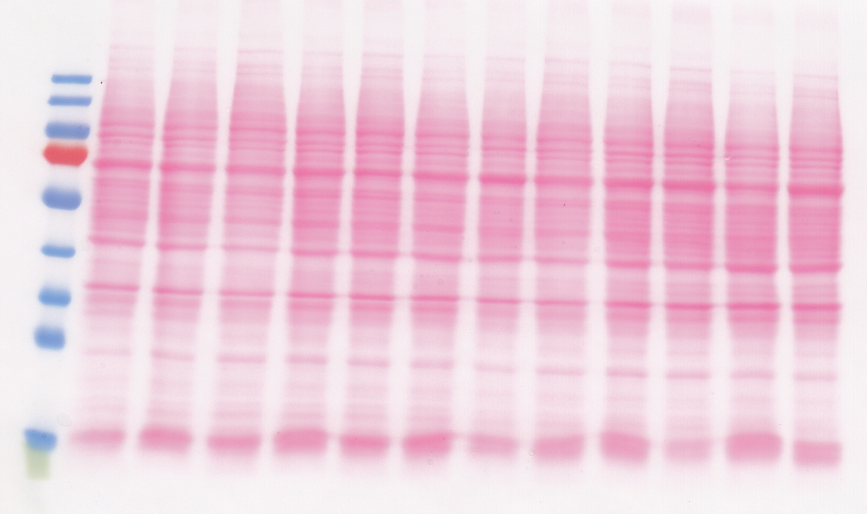
**

**Figure S12b:** Uncropped Ponceau S-stained membrane used for

detection occludin expression (**Figure S12a).** Of note, the same membrane

was used for blots shown in **Figures S10a** and **S13a**.

—65 kDa

**Figure S13a:** Uncropped Western blot of occludin expression.

Area that is outlined in blue is shown in the middle panel of Fig. 4**f**.

**
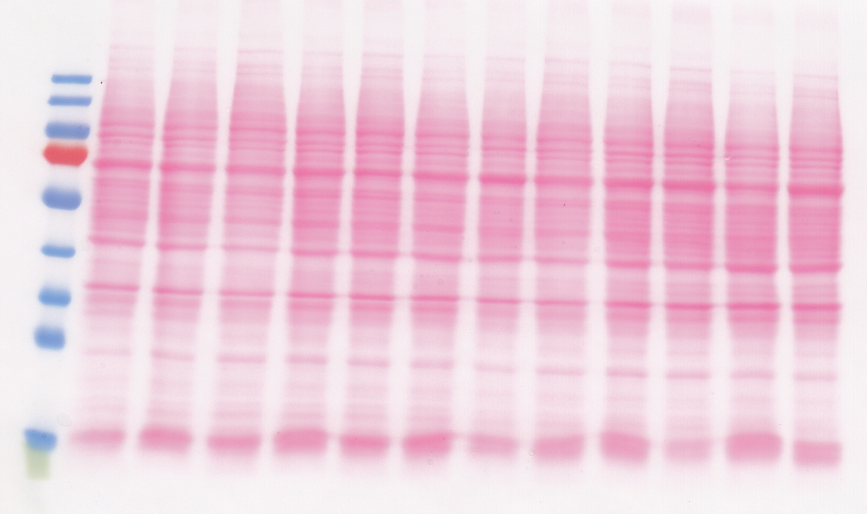
**

**Figure S13b:** Uncropped Ponceau S-stained membrane used for

detection occludin expression (**Figure S13a).** Of note, the same membrane

was used for blots shown in **Figures S10a** and **S12a**.

—65 kDa

**Figure S14a:** Uncropped Western blot of occludin expression.

Area that is outlined in blue is shown in the lower panel of Fig. 4**f**.

**
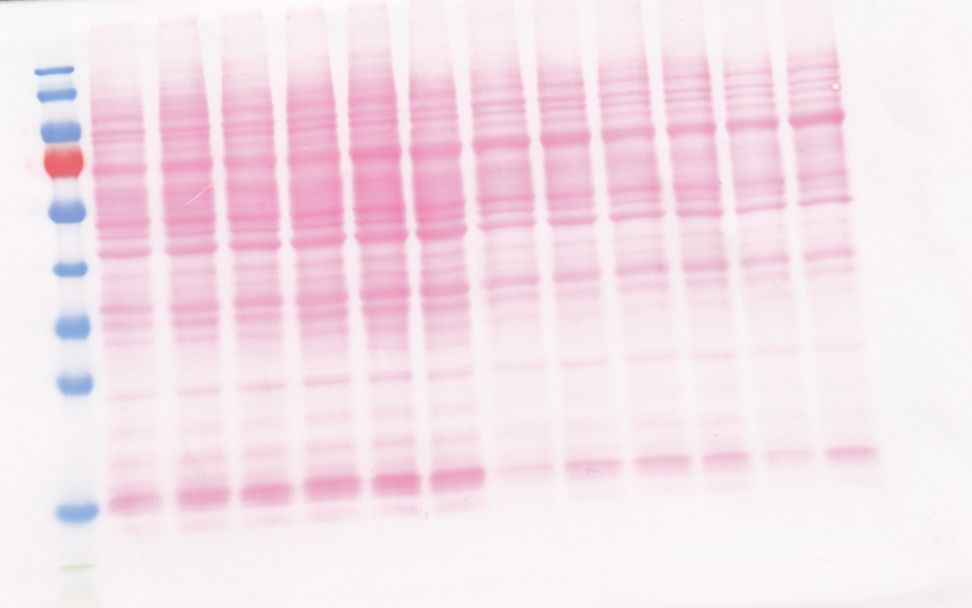
**

**Figure S14b:** Uncropped Ponceau S-stained membrane used for

detection of occludin expression. (**Figure S14a**).

—135 kDa

**Figure S15a:** Uncropped Western blot of E-cadherin expression.

Area that is outlined in blue is shown in the upper panel of Fig. 4**i**.


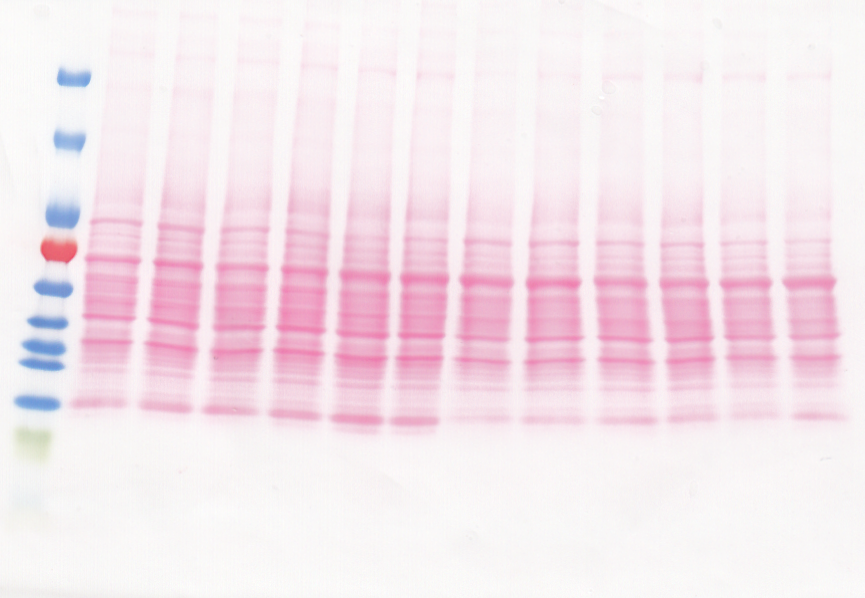


**Figure S15b:** Uncropped Ponceau S-stained membrane used for

detection of E-cadherin expression. (**Figure S15a)**.

—135 kDa

**Figure S16a:** Uncropped Western blot of E-cadherin expression.

Area that is outlined in blue is shown in the middle panel of Fig. 4**i**.


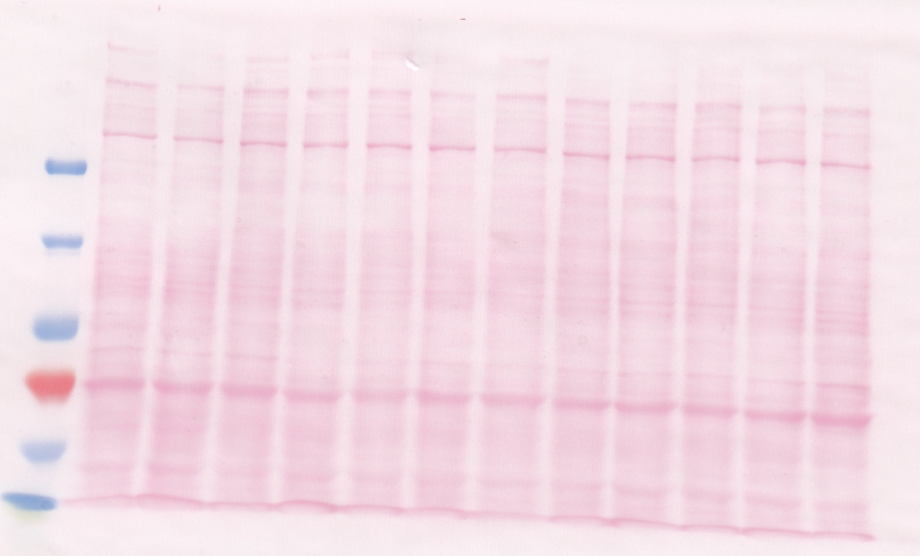


**Figure S16b:** Uncropped Ponceau S-stained membrane used for

detection of E-cadherin expression. (**Figure S16a)**.

—135 kDa

**Figure S17a:** Uncropped Western blot of E-cadherin expression.

Area that is outlined in blue is shown in the lower panel of Fig. 4**i**.


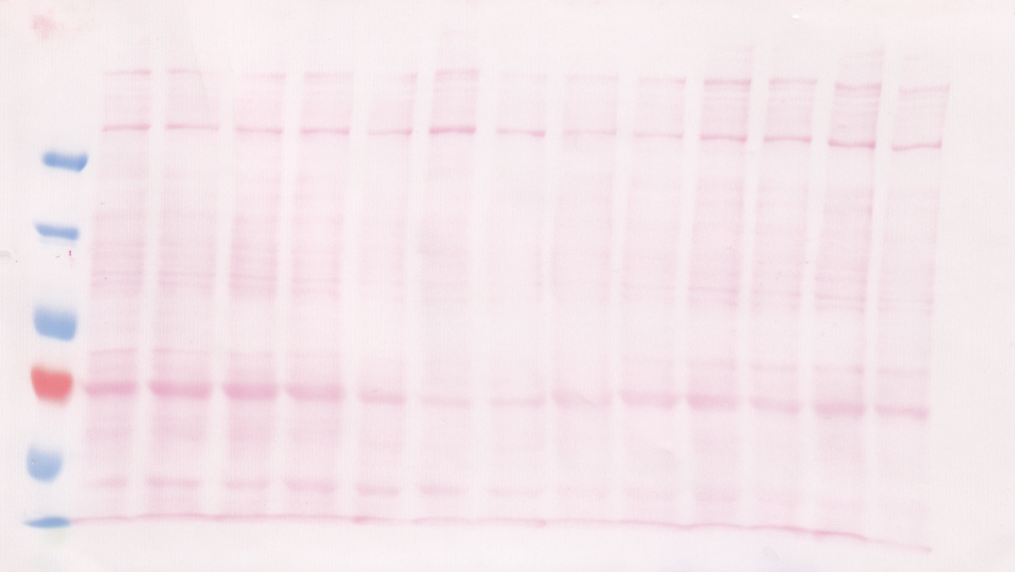


**Figure S17b:** Uncropped Ponceau S-stained membrane used for

detection of E-cadherin expression. (**Figure S17a)**.
